# Density scaling of phantom materials for a 3D dose verification system

**DOI:** 10.1002/acm2.12357

**Published:** 2018-05-21

**Authors:** Kensuke Tani, Yukio Fujita, Akihisa Wakita, Ryohei Miyasaka, Ryuzo Uehara, Takumi Kodama, Yuya Suzuki, Ako Aikawa, Norifumi Mizuno, Jiro Kawamori, Hidetoshi Saitoh

**Affiliations:** ^1^ Department of Radiological Sciences Graduate School of Tokyo Metropolitan University Arakawa Japan; ^2^ Department of Radiation Oncology Tokai University School of Medicine Isehara Japan; ^3^ Department of Radiation Oncology National Cancer Center Hospital Tsukiji Japan; ^4^ Department of Radiation Oncology Chiba Cancer Center Chiba Japan; ^5^ Department of Radiation Oncology National Cancer Center Hospital East Kashiwa Japan; ^6^ Department of Radiation Oncology Saitama Cancer Center Ina Japan; ^7^ Department of Radiation Oncology Tokyo Dental College Ichikawa General Hospital Ichikawa Japan; ^8^ Department of Radiation Oncology St. Luke's International Hospital Tokyo Japan

**Keywords:** Delta4, density scaling, dose verification, IMRT, phantom

## Abstract

In this study, the optimum density scaling factors of phantom materials for a commercially available three‐dimensional (3D) dose verification system (Delta4) were investigated in order to improve the accuracy of the calculated dose distributions in the phantom materials. At field sizes of 10 × 10 and 5 × 5 cm^2^ with the same geometry, tissue‐phantom ratios (*TPR*s) in water, polymethyl methacrylate (PMMA), and Plastic Water Diagnostic Therapy (PWDT) were measured, and *TPR*s in various density scaling factors of water were calculated by Monte Carlo simulation, Adaptive Convolve (AdC, Pinnacle^3^), Collapsed Cone Convolution (CCC, RayStation), and AcurosXB (AXB, Eclipse). Effective linear attenuation coefficients (*μ*_eff_) were obtained from the *TPR*s. The ratios of *μ*_eff_ in phantom and water ((*μ*_eff_)_pl,water_) were compared between the measurements and calculations. For each phantom material, the density scaling factor proposed in this study (*DSF*) was set to be the value providing a match between the calculated and measured (*μ*_eff_)_pl,water_. The optimum density scaling factor was verified through the comparison of the dose distributions measured by Delta4 and calculated with three different density scaling factors: the nominal physical density (PD), nominal relative electron density (ED), and *DSF*. Three plans were used for the verifications: a static field of 10 × 10 cm^2^ and two intensity modulated radiation therapy (IMRT) treatment plans. *DSF* were determined to be 1.13 for PMMA and 0.98 for PWDT. *DSF* for PMMA showed good agreement for AdC and CCC with 6 MV x ray, and AdC for 10 MV x ray. *DSF* for PWDT showed good agreement regardless of the dose calculation algorithms and x‐ray energy. *DSF* can be considered one of the references for the density scaling factor of Delta4 phantom materials and may help improve the accuracy of the IMRT dose verification using Delta4.

## 1 INTRODUCTION

It is necessary to verify the agreement between dose distributions calculated by a radiation treatment planning system (RTPS) and delivered by a linear accelerator (linac) for intensity modulated radiation therapy (IMRT).^1^, ^2^ A number of approaches and systems have been developed for this verification.^2^, ^3^, ^4^, ^5^, ^6^, ^7^, ^8^, ^9^, ^10^, ^11^, ^12^, ^13^ Recently, three‐dimensional (3D) dose verification systems consisting of a solid phantom and detector arrays have become commercially available. These 3D dose verification systems can measure the absorbed dose at thousands of measurement points, and they are efficient in verifying the 3D dose distribution.

IMRT dose verifications should be evaluated more accurately. Kly et al.^14^ showed that institutional patient‐specific IMRT quality assurance (QA) does not necessarily detect unacceptable plans. In their study, 14% of plans accepted by institutional IMRT QA were described as fail by an audit. In other words, even if an IMRT plan is accepted by one verification system, this does not ensure that the plan will be accepted by another verification system. Although various causes can be considered for this discrepancy, commissioning of verification systems is important to ensure the evaluation certainty. Because the verification is basically comparing the dose distributions in a solid phantom measured by the detectors and that calculated by the RTPS, the appropriate density scaling factor of the solid phantom used in the verification system should be adopted in the RTPS, where the density scaling factor is defined as a density to be assigned for the phantom material in RTPS (e.g., physical density, relative electron density, or other value).

**Figure 1:**
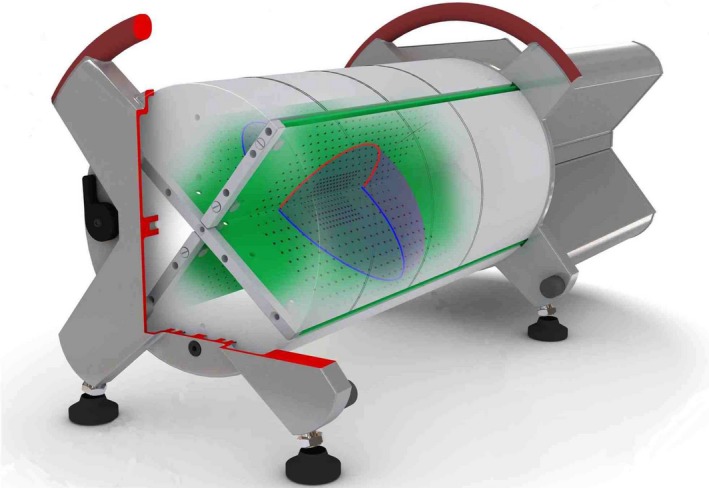
Appearance of a 3D dose verification system (Delta4, ScandiDos). The silicon diodes are placed at 5 mm intervals in a central 6 cm × 6 cm area and at 10 mm intervals elsewhere in a 20 cm × 20 cm area on two orthogonal boards in the cylindrical phantom materials with a diameter of 22 cm. The standard detector geometry is depicted (“X”). If the attachment for sagittal‐coronal option was used, the detector geometry could be rotated to the “+” orientation.

A number of studies for IMRT 3D dose verification systems have been reported. One of these 3D dose verification systems (Delta4 (ScandiDos, Inc., Ashland, VA, USA)) consists of 1069 silicon diodes arranged on two orthogonal boards in polymethyl methacrylate (PMMA) or Plastic Water Diagnostic Therapy (PWDT) as shown in Fig. 1. It has been used for IMRT patient‐specific QA,^13^, ^15^, ^16^, ^17^, ^18^, ^19^, ^20^ commissioning of volumetric modulated arc therapy (VMAT),^21^ and comparisons of dose calculation algorithms.^22^, ^23^ However, these studies had an approximately 2% dose difference resulting from the difference in density scaling factors of the phantom materials. Pham et al.^15^ and Feygelman et al.^18^ evaluated Delta4 with the same photon energy, phantom material, and RTPS; the former adopted a density scaling factor of 1.19, while the latter adopted 1.14 for the PMMA phantom. Other studies^13^, ^17^, ^19^, ^21^, ^22^, ^23^ have not reported the adopted density scaling factor, and their appropriateness has not been discussed so far.

The purpose of this study was to clarify the optimum density scaling factor for PMMA and PWDT in order to improve the accuracy of the calculated dose distributions in the phantom materials of Delta4. The density scaling factors proposed in this study (*DSF*) for PMMA and PWDT were determined from measurements and calculations with several algorithms. The appropriateness of the *DSF* was validated by dose verifications with several plans using commercially available algorithms.

## 2 MATERIALS AND METHODS

### 2.A Effective linear attenuation coefficients (*μ*_eff_)

#### 2.A.1 Measurements of *μ*_eff_

**Figure 2:**
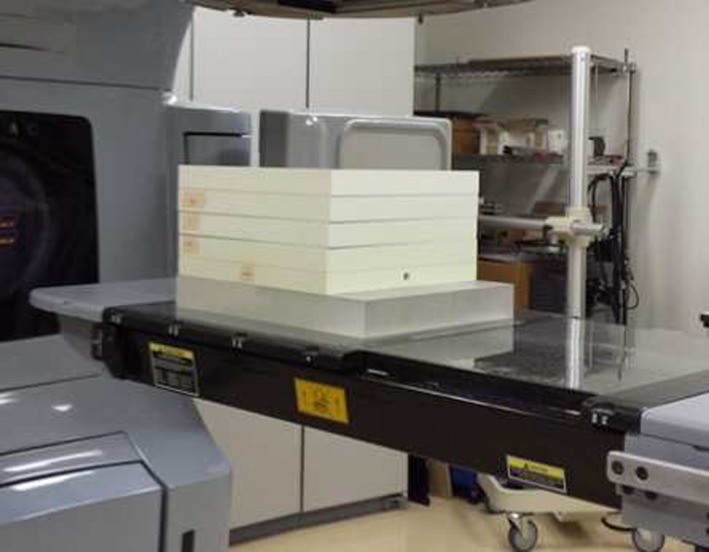
Example of the phantom geometry used to measure *TPR*s. Normally, the manufacturer provides one slab for buildup, one slab for chamber insert, and one slab for backscatter in order to measure the absorbed dose at a depth of 4.25 cm in PMMA and 4.95 cm in PWDT for the cross‐calibration of Delta4. This figure shows four PWDT buildup slabs, one PWDT chamber insert slab (SCD = 100 cm, depth = 15.45 cm), and one PMMA slab for backscatter. The manufacture provides only PMMA for the backscatter slab. In this study, several sets of the calibration slab phantoms were stacked to measure *TPR*s at several depths. To calculate *TPR*s in the Monte Carlo simulation and RTPS, the geometry and phantoms were modeled the same as the geometry of the measurements.

Tissue‐phantom ratios (*TPR*s) in water, PMMA, and PWDT were measured at field sizes of 10 × 10 and 5 × 5 cm^2^ with 6 and 10 MV x rays from linacs (Clinac iX and 21EX (Varian Medical Systems, Palo Alto, CA, USA)). Calibration slab phantoms of Delta4 were stacked, and an ionization chamber (30013, PTW, Freiburg, Germany) was set at a source‐to‐chamber distance (SCD) of 100 cm, as shown in Fig. 2. The depths (*d*) were 4.25, 7.05, 9.25, and 12.05 cm in PMMA and 4.95, 8.45, 11.95, and 15.45 cm in PWDT. The reference depth of the *TPR*s was set to the shallowest depth. The *μ*_eff_ at each condition were determined from the slope of the exponential regression curve approximating the *TPR* curve.

#### 2.A.2 Calculations of *μ*_eff_

The following dose calculation algorithms were used to calculate the *TPR*s: Monte Carlo (the EGSnrc Monte Carlo code system^24^, ^25^ and BEAMnrc code system^26^), Adaptive Convolve (AdC) (Pinnacle^3^ ver. 9.10, Philips Radiation Oncology Systems, Fitchburg, WI, USA), Collapsed Cone Convolution (CCC) (RayStation ver. 4.5, RaySearch Laboratories, Stockholm, Sweden), and AcurosXB (AXB) (Eclipse ver. 11, Varian Medical Systems, Palo Alto, CA, USA).

The geometries of the phantoms in the calculations were modeled to be the same as the measurement. The phantoms were assigned as water but the physical densities were varied from 0.96 to 1.19 g/cm^3^. The reasons for assigning the material of the phantoms as water were: (a) the phantoms could not be assigned as PMMA for AdC and PWDT for AdC, CCC, and AXB, and (b) the physical density of the materials other than water could not be changed from the default physical density for CCC and AXB. For the Monte Carlo simulation, the phantom materials were generated in PEGS (Preprocessor for EGS).^27^ The phase space data of the particles were scored and validated by comparing between calculated and measured depth dose and off‐axis ratio in water, and they were used in all simulations. The simulations were repeated until a statistical uncertainty of less than 0.1% was obtained. For the RTPS dose calculations, the grid size was 2 mm for AXB and 1 mm for the other dose calculation algorithms because dose using AXB with a grid size of 1 mm could not be calculated under several conditions due to a shortage of computer memory resources. The reference depth of the *TPR*s was set to the shallowest depth. The *μ*_eff_ at each condition were determined from the slope of the exponential regression curve approximating the *TPR* curve.

### 2.B Determining *DSF*

*DSF*s were determined through comparisons of the measured and calculated *TPR*s. To compare the *TPR*s between the measurements and calculations, the ratios of *μ*_eff_ in phantom and water ((*μ*_eff_)_pl,water_) were used. The measured (*μ*_eff_)_pl,water_ were obtained by dividing the *μ*_eff_ measured in phantoms by the *μ*_eff_ measured in water. The calculated (*μ*_eff_)_pl,water_ were obtained by dividing the *μ*_eff_ calculated with various density scaling factors by the *μ*_eff_ calculated with the density scaling factor of 1.0. Although the beam qualities of the linacs used in this study were consistent, the *μ*_eff_ calculated with the density scaling factor of 1.0 had a small variation among the dose calculation algorithms due to the modeling accuracy. This normalization is to make the changes of the slope of the *TPR*s for density scaling factors independent of the modeling accuracy of the each dose calculation algorithm.

The measured (*μ*_eff_)_pl,water_ were used as the reference value in the comparisons. The calculated (*μ*_eff_)_pl,water_ were obtained as a function of the density scaling factors. The regression line approximating the median values of the (*μ*_eff_)_pl,water_ calculated by the dose calculation algorithms for several density scaling factors was drawn. When the regression line matched the measured (*μ*_eff_)_pl,water_, the density scaling factor was set to *DSF*_regression_ for the x‐ray energy, field size, and phantom material. Finally, for each phantom material, the mean value of *DSF*_regression_ was used to define *DSF*.

Additionally, specific *DSF*s for dose calculation algorithms (*sDSF*) were determined. Individually, the regression line approximating the (*μ*_eff_)_pl,water_ calculated by each dose calculation algorithm for several density scaling factor was drawn. When each regression line matched the measured (*μ*_eff_)_pl,water_, the density scaling factor was set to *sDSF* of the dose calculation algorithm for a given condition.

### 2.C Dose verifications with different density scaling factors

For each phantom material, the appropriateness of *DSF* was verified through comparisons of the dose distributions measured with Delta4 and calculated with three different density scaling factors: the nominal physical density (PD), nominal relative electron density (ED), and *DSF*. The PDs of PMMA and PWDT are 1.190 and 1.039 g/cm^3^, and EDs of PMMA and PWDT are 1.159 and 1.003, respectively.^28^, ^29^ The following dose calculation algorithms were used for the verification: AdC (Pinnacle^3^ ver. 9.0 for PMMA and 9.10 for PWDT), CCC, and AXB. Three plans were used for the verifications: one was 10 × 10, which denotes a static field of 10 × 10 cm^2^ with static gantry angles of 45° and 315° for the “+” (sagittal‐coronal option) detector geometry and 0° for the “X” (standard) detector geometry. The others were IMRT plans using “mock head&neck” and “mock prostate” in AAPM TG‐119.^30^ The IMRT plans were created following the dose constraints shown in AAPM TG‐119.^30^ The delivery techniques for the IMRT plans were step‐and‐shoot in Pinnacle^3^ and VMAT in the others. Before all measurements, the dose per monitor unit (DMU) of each x‐ray energy was obtained in accordance with the standard dosimetry protocol, and the daily machine output was corrected by the daily correction factor from the built‐in Delta4 software. These dose verifications were evaluated according to the pass rate of the global gamma index (gGI) for different criteria (2%/2 mm and 1%/1 mm) and the median of the global dose deviation (gDD) with the lower dose threshold of 20%. The normalization doses for the gGI and gDD were set to the measured dose at the isocenter for 10 × 10 and 2.0 Gy for the IMRT plans.

## 3 RESULTS

### 3.A *μ*_eff_ of *TPR*s

**Figure 3:**
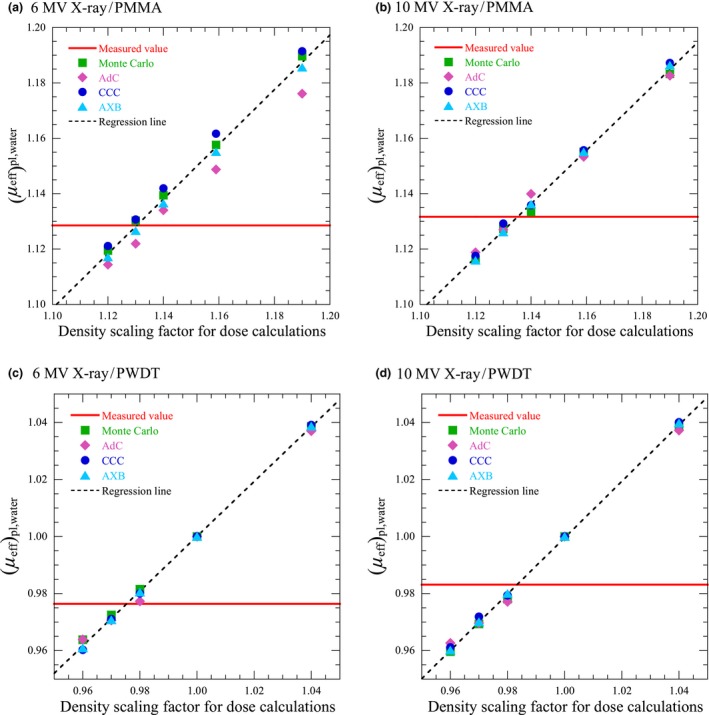
Calculated (*μ*_eff_)_pl,water_ as a function of density scaling factor for dose calculation and measured (*μ*_eff_)_pl,water_, presented as a horizontal line (field size = 10 × 10 cm^2^). The regression line approximating the median values of the calculated (*μ*_eff_)_pl,water_ of the dose calculation algorithms at each density scaling factor is represented as a diagonal dashed line.

Figure 3 shows the measured (*μ*_eff_)_pl,water_ and changes in the calculated (*μ*_eff_)_pl,water_ for density scaling factors at a field size of 10 × 10 cm^2^. The measured (*μ*_eff_)_pl,water_ were obtained within only the actual phantoms with no change in density, hence they are drawn as a horizontal line. The measured (*μ*_eff_)_pl,water_ at 6 and 10 MV were 1.13 and 1.13 for PMMA, and 0.98 and 0.98 for PWDT, respectively.

**Figure 4:**
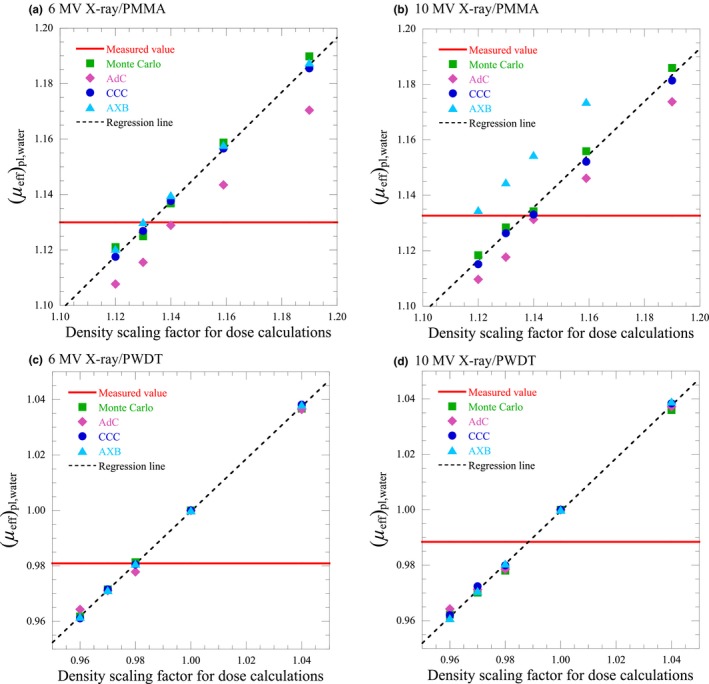
Calculated (*μ*_eff_)_pl,water_ as a function of density scaling factor for dose calculation and measured (*μ*_eff_)_pl,water_, represented as a horizontal line (field size = 5 × 5 cm^2^). The regression line approximating the median values of the calculated (*μ*_eff_)_pl,water_ of the dose calculation algorithms at each density scaling factor is represented as a diagonal dashed line.

Figure 4 shows the measured (*μ*_eff_)_pl,water_ and changes in the calculated (*μ*_eff_)_pl,water_ for density scaling factors at a field size of 5 × 5 cm^2^. The measured (*μ*_eff_)_pl,water_ at 6 and 10 MV were 1.13 and 1.13 for PMMA, and 0.98 and 0.99 for PWDT, respectively.

### 3.B *DSF* of PMMA and PWDT

Based on Figs. 3 and 4, *DSF*_regression_ was determined as the density scaling factor when the regression line matched the measured (*μ*_eff_)_pl,water_. At a field size of 10 × 10 cm^2^, *DSF*_regression_ of 6 and 10 MV x ray were 1.13 and 1.14 for PMMA, and 0.98 and 0.98 for PWDT, respectively. At a field size of 5 × 5 cm^2^, *DSF*_regression_ of 6 and 10 MV x ray were 1.13 and 1.14 for PMMA, and 0.98 and 0.99 for PWDT, respectively. Therefore, *DSF* in this study was determined as 1.13 for PMMA and 0.98 for PWDT, as given in Table 1.

**Table 1:** Nominal physical density, nominal relative electron density, and *DSF* of PMMA and PWDT

Phantom material	Physical density [g cm^−3^]	Relative electron density	*DSF*
PMMA	1.190	1.159	1.13
PWDT	1.039	1.003	0.98

At a field size of 10 × 10 cm^2^, *sDSF* of Monte Carlo, AdC, CCC, and AXB for PMMA were 1.13, 1.13, 1.15, and 1.12 in 6 MV x ray, and 1.13, 1.13, 1.14, and 1.13 in 10 MV x ray, respectively. The *sDSF* of Monte Carlo, AdC, CCC, and AXB for PWDT were 0.97, 0.98, 0.98, and 0.97 in 6 MV x ray, and 0.98, 0.99, 0.98, and 0.98 in 10 MV x ray, respectively. At a field size of 5 × 5 cm^2^, *sDSF* of Monte Carlo, AdC, CCC, and AXB for PMMA were 1.13, 1.14, 1.15, and 1.11 in 6 MV x ray, and 1.13, 1.13, 1.12, and 1.11 in 10 MV x ray, respectively. The *sDSF* of Monte Carlo, AdC, CCC, and AXB for PWDT were 0.97, 0.98, 0.99, and 0.98 in 6 MV x ray, and 0.99, 0.98, 0.99, and 0.98 in 10 MV x ray, respectively.

### 3.C Dose verifications with different density scaling factors

**Figure 5:**
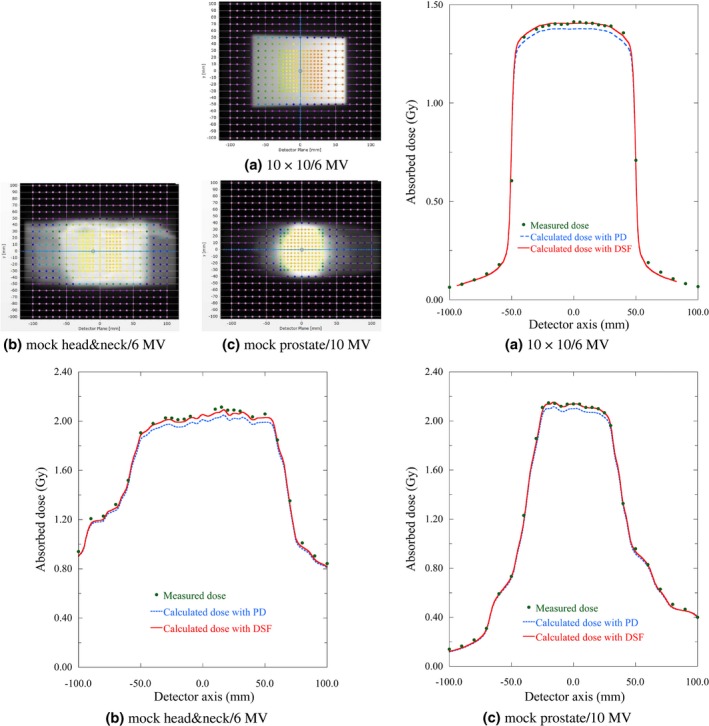
Comparisons between the measured and calculated dose profiles in the Delta4 PMMA phantom with AdC for the several dose verifications using PD and *DSF*. The pictures at the upper left show the measured dose distributions for each plan on one of the two orthogonal detector boards and the blue lines on the pictures show the position of the displayed profiles. [Correction added on 28 May, after first Online publication: Figure 5 position rearrangement has been updated.]

**Figure 6:**
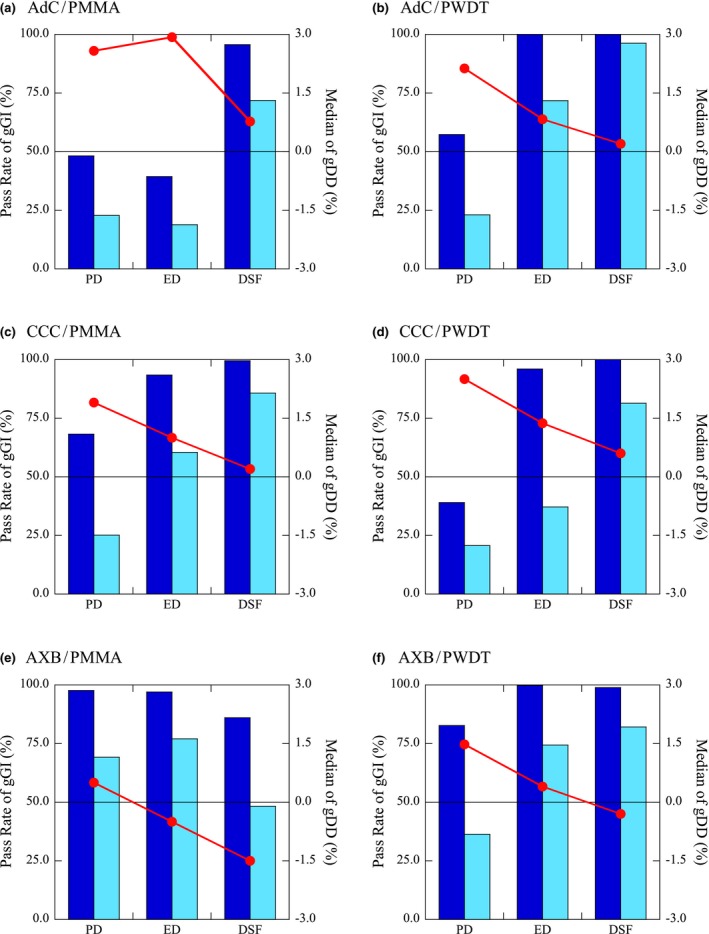
Pass rates of gGI (left axis, bars; 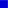: gGI with the criterion of 2%/2 mm, and 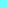: gGI with the criterion of 1%/1 mm) and median of gDD (right axis, circle and line; 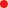) of several dose calculation algorithms with physical density (PD), relative electron density (ED), and *DSF* (Plan: 10 × 10, x‐ray energy: 6 MV).

Figure 5 shows the measured and calculated dose profiles in Delta4 PMMA phantom with AdC for the several dose verifications using PD and *DSF*. These verification plans were 10 × 10 with 6 MV x ray, mock head&neck with 6 MV x ray, and mock prostate with 10 MV x ray. Fig. 6 shows the pass rates of gGI and median of gDD of the dose calculation algorithms with different density scaling factors for the dose verifications of 10 × 10 with 6 MV x ray. Tables 2 and 3 show the summary of the pass rates of gGI and median of gDD of the dose calculation algorithms with different density scaling factors for the dose verifications of 10 × 10 and IMRT within PMMA and PWDT.

**Table 2:** Summary of the pass rates (%) of gGI with the criteria of 2%/2 mm and 1%/1 mm, and median (%) of gDD of several dose calculation algorithms with physical density (PD), relative electron density (ED), and *DSF* for PMMA

Plan/x‐ray energy	AdC			CCC			AXB		
Metrics	PD	ED	DSF	PD	ED	DSF	PD	ED	DSF
10 × 10/6 MV									
gGI (2%/2 mm)	48.3	39.4	95.6	68.2	93.4	99.5	97.7	97.0	86.1
gGI (1%/1 mm)	22.8	18.9	71.9	25.2	60.4	85.7	69.2	77.0	48.3
Median of gDD	2.6	2.9	0.8	1.9	1.0	0.2	0.5	−0.5	−1.5
Mock head&neck/6 MV									
gGI (2%/2 mm)	67.2	54.4	98.2	85.6	96.2	99.5	64.5	83.0	97.0
gGI (1%/1 mm)	31.9	27.8	81.8	31.9	27.8	81.8	45.2	59.3	77.8
Median of gDD	2.1	2.4	0.5	1.6	0.9	0.2	1.8	1.1	0.3
Mock prostate/6 MV									
gGI (2%/2 mm)	79.8	72.7	100.0	93.5	98.7	98.9	87.6	97.9	99.2
gGI (1%/1 mm)	51.3	46.7	87.7	67.3	88.1	89.2	63.7	82.9	92.2
Median of gDD	1.7	1.9	0.1	1.3	0.5	−0.3	1.2	0.5	−0.1
10 × 10/10 MV									
gGI (2%/2 mm)	63.4	52.7	98.7	99.0	99.7	96.0	97.5	94.7	83.8
gGI (1%/1 mm)	21.6	19.4	75.8	82.0	82.1	61.8	81.7	64.8	38.2
Median of gDD	2.3	2.5	0.7	0.5	−0.2	−0.9	−0.2	−0.9	−1.7
Mock head&neck/10 MV									
gGI (2%/2 mm)	83.2	71.8	99.8	95.2	99.1	99.1	78.7	93.1	98.8
gGI (1%/1 mm)	37.2	33.3	89.3	71.1	84.1	85.7	54.5	71.3	82.6
Median of gDD	1.7	1.9	0.5	0.9	0.4	−0.1	1.3	0.7	0.3
Mock prostate/10 MV									
gGI (2%/2 mm)	88.1	82.3	100.0	99.4	99.0	93.4	95.5	98.7	99.0
gGI (1%/1 mm)	57.5	53.7	97.2	89.6	84.6	73.7	78.8	88.8	91.1
Median of gDD	1.5	1.7	0.3	0.2	−0.4	−0.9	0.6	0.1	−0.3

**Table 3:** Summary of the pass rates (%) of gGI with the criteria of 2%/2 mm and 1%/1 mm, and median (%) of gDD of several dose calculation algorithms with physical density (PD), relative electron density (ED), and *DSF* for PWDT

Plan/x‐ray energy	AdC			CCC			AXB		
Metrics	PD	ED	DSF	PD	ED	DSF	PD	ED	DSF
10 × 10/6 MV									
gGI (2%/2 mm)	57.3	100.0	100.0	39.1	96.0	100.0	82.7	99.8	98.9
gGI (1%/1 mm)	23.1	71.7	96.3	20.7	37.2	81.4	36.4	74.4	82.1
Median of gDD	2.1	0.8	0.2	2.5	1.4	0.6	1.5	0.4	−0.3
Mock head&neck/6 MV									
gGI (2%/2 mm)	74.7	90.1	96.0	73.4	92.8	98.8	62.4	78.7	91.4
gGI (1%/1 mm)	53.4	63.8	69.8	52.7	70.0	81.8	42.7	54.5	68.2
Median of gDD	1.4	0.6	0.1	2.1	1.2	0.7	2.1	1.3	0.9
Mock prostate/6 MV									
gGI (2%/2 mm)	74.7	90.1	96.5	79.4	97.2	99.9	72.5	91.9	98.6
gGI (1%/1 mm)	53.4	63.8	70.3	58.5	78.1	90.8	48.3	63.9	79.9
Median of gDD	1.4	0.6	0.1	1.9	0.9	0.2	1.9	1.3	0.7
10 × 10/10 MV									
gGI (2%/2 mm)	99.0	100.0	100.0	96.9	100.0	100.0	84.0	99.8	100.0
gGI (1%/1 mm)	43.2	96.9	95.2	46.6	93.7	97.1	36.0	69.3	87.1
Median of gDD	1.4	0.4	−0.1	1.3	0.5	0.0	1.4	0.7	0.2
Mock head&neck/10 MV									
gGI (2%/2 mm)	90.3	99.4	100.0	84.7	95.2	98.5	63.8	78.3	87.3
gGI (1%/1 mm)	62.5	78.3	83.2	56.9	75.9	85.8	37.6	48.2	60.3
Median of gDD	1.1	0.3	0.1	1.7	1.0	0.6	2.2	1.5	1.1
Mock prostate/10 MV									
gGI (2%/2 mm)	90.3	99.4	100.0	94.9	99.7	100.0	76.6	90.5	96.6
gGI (1%/1 mm)	62.5	78.3	83.7	80.0	93.6	93.9	53.2	67.8	78.4
Median of gDD	1.1	0.3	0.1	1.1	0.3	−0.1	1.9	1.3	1.0

Figure 6 shows the graphs of the numerical data of 10 × 10 with 6 MV x ray in Tables 2 and 3. The pass rates of gGI and median of gDD were changed dramatically according to the changes of adopted density scaling factors. For AdC (ver.9.0) in PMMA, there was an unreasonable change at the ED. The measured dose distributions were consistent regardless of the adopted density scaling factors in the dose verifications. The calculated dose distributions should change according to the adopted density scaling factor; the calculated dose should become gradually higher when the adopted density scaling factor becomes gradually lower. However, the median of gDD using ED (1.159) was not between that using PD (1.19) and *DSF* (1.13). This may be due to the coarse resolution of the mass attenuation coefficient in an older version of Pinnacle^3^, as pointed out by Dickof.^31^ Except for AXB within PMMA, the pass rates of gGI increased and median of gDD moved close to 0% from the PD to *DSF*. The tendency was consistent regardless of the dose calculation algorithms, verification plans, and x‐ray energy within PWDT, as shown in Table 3. *DSF* for PWDT showed good agreement between the measured and calculated dose distributions under multiple conditions. On the other hand, as shown in Table 2, *DSF* for PMMA showed good agreement between those in AdC and CCC with 6 MV x ray, and AdC for 10 MV x ray for 10 × 10. The PD or ED showed good agreement between those in AXB with 6 MV x ray, and CCC and AXB with 10 MV x ray for 10 × 10. The results within PMMA varied depending on the dose calculation algorithms and x‐ray energy. Although the dose verifications of 10 × 10 for AXB within PMMA were conducted in three institutions after the absolute dose calibration for the Delta4 detectors, these results were unchanged. This removes the dependence of these results on site‐specific errors such as linac output, cross‐calibration of Delta4, or beam data in RTPS.

In Tables 2 and 3, the results for CCC within PMMA and PWDT were obtained at one institution, and those for AXB within PMMA and PWDT were obtained at a different institution. Although the results of the dose verifications for CCC within PWDT were consistent for both 6 and 10 MV x ray, the results for CCC within PMMA were not consistent. The optimum density scaling factor for CCC in PMMA was *DSF* in 6 MV x ray and ED in 10 MV x ray. Although the results of mock head&neck for CCC with 10 MV x ray were slightly different from those of 10 × 10 and mock prostate in PMMA, it was the same tendency as that seen in the results in PWDT. However, the optimum density scaling factor of PMMA for AXB seemed to be PD or ED in 10 × 10 and *DSF* in IMRT. The reason may be the systematic dose difference of 1% for the IMRT plans between the linac output and dose calculation in the institution. The results of the IMRT verifications in PWDT were also higher by 1% compared to 10 × 10.

## 4 DISCUSSION

The choice of density scaling factor has a large effect on the ability to accurately calculate dose distributions in the Delta4 phantoms. This is evidenced by the fact that the average difference of the pass rates of gGI with the criterion of 2%/2 mm between PD and *DSF* for mock head&neck plans in 6 MV x ray were 25.8% in PMMA and 25.2% in PWDT. Furthermore, as shown in Fig. 5, the calculated dose profiles adopted with PD were clearly different from both the measured dose distributions and the calculated dose profiles adopted with *DSF*. The choice of density scaling factor plays a crucial role not only for patient‐specific IMRT QA in order to appropriately judge for pass or fail, but also for the IMRT commissioning for RTPS. The reason why the choice is important for the IMRT commissioning is that the calculated dose distributions would need to be adjusted by modifying several dose calculation parameters (e.g., dosimetric leaf gap, MLC transmission, tongue and groove effect, or focal spot size) compared with measured dose distributions.^32^, ^33^, ^34^, ^35^ For these modifications, the usage of a solid phantom with detector arrays or films would be inevitable in order to evaluate steep dose distributions. If the dose verifications for these modifications were conducted with an inadequate density scaling factor, these parameters may be decided as inadequate values. Therefore, the appropriate choice of the density scaling factor to improve the accuracy of the calculated dose distribution in solid phantoms is important for patient‐specific IMRT QA and IMRT commissioning for RTPS.

Although the relative electron density is commonly assigned to solid phantoms in RTPS, its appropriateness for density scaling has been demonstrated for the equivalent path length in narrow photon beams.^36^ However, the optimum density scaling factor for 3D dose verification is not the ratio for the equivalent path length of photon interactions but the density in RTPS that gives a calculated dose distribution closely matching the measured dose distribution. Therefore, the *DSF*s were obtained according to the changes of depth dose that included the component of the scattered dose in the phantoms. To determine the cause for this divergence as the component of the scattered dose in the phantom or others, further study should be conducted with more types of solid phantoms and geometry conditions. In this study for the density scaling factors of Delta4 phantom materials, *DSF* were obtained as lower values than the nominal relative electron densities through our original method.

Regarding the density scaling factors of PMMA applied in other studies of Delta4, Pham and Luo^15^ used 1.19 in Pinnacle^3^. Geurts et al. used 1.19 in TomoTherapy system.^20^ Kumagai et al.^37^ found 1.16 for 4 MV and 1.15 for 10 MV in Pinnacle^3^. Feygelman et al.^18^ used 1.14 in Pinnacle^3^ and their value was close to the *DSF* in the previous studies. TG‐21^38^ was the reference for the relative electron density of 1.14. TG‐21^38^ calculated a relative electron density of 1.137 from the physical density of 1.17 g/cm^3^ for PMMA. However, the physical density of PMMA was shown as 1.19 g/cm^3^.^28^, ^29^, ^39^ In additional investigations, we measured the physical density and analyzed the elemental composition of PMMA in a portion of Delta4 with the thermal conductivity method for hydrogen and carbon, and the infrared absorption spectrophotometry for oxygen. Consequently, the physical density was 1.19 g/cm^3^, and the elemental composition closely matched the nominal elemental composition.^28^, ^29^, ^39^ The *DSF* are the lowest density scaling factor acquired theoretically.

*DSF* was shown to be the optimum density scaling factor for PWDT regardless of the dose calculation algorithm and x‐ray energy. Furthermore, the changes of the (*μ*_eff_)_pl,water_ calculated by the dose calculation algorithms were consistent at each density scaling factor for PWDT. On the other hand, these changes were not consistent for PMMA. Specifically, these changes of AdC were lower than those of other dose calculation algorithms and those of AXB were higher in several conditions, as shown in Figs. 3 and 4. The reason these differences occurred in treating different densities of water for PMMA was difficult to specify because the details of the dose calculation algorithms related to treat different densities of water in the calculations opened to the public were limited. At least, the results showed the possibility that optimum density scaling factor for AdC may become higher than *DSF* such as ED and the one for AXB may become lower than *DSF* through the dose verifications. However, in the dose verifications for PMMA, *DSF* was the optimum density scaling factor in AdC and CCC with 6 MV x ray, and AdC with 10 MV x ray. The PD or ED may be the optimum density scaling factor in AXB with 6 MV x ray, and CCC and AXB with 10 MV x ray, nevertheless none of the (*μ*_eff_)_pl,water_ calculated by the dose calculation algorithms matched the measured (*μ*_eff_)_pl,water_ at PD and ED, as shown in Fig. 2. A reason for the considerable deviation may be the accuracy of the absorbed dose calculation in a higher density of water. Because *DSF* were obtained from the slopes of the *TPR*s, the *DSF* were not found to be an appropriate density scaling factor for the absorbed dose calculation in different densities of water. If there was some mismatch or uncertainty between the slope of the depth dose and absorbed dose calculated in a different density of water, it should be corrected by something other than the density scaling factor.

## 5 CONCLUSIONS

The difference in density scaling factors caused a bigger dosimetric difference than the pass/fail criterion. We clarified *DSF* of PMMA and PWDT from measurements and calculations, and validated the appropriateness of *DSF*. The *DSF* were lower than not only the PD but also the ED. *DSF* can be used as a reference for the density scaling factor of the Delta4 phantom material in multiple clinical institutions and may help improve the accuracy of the IMRT dose verification using Delta4.

## CONFLICT OF INTEREST

The authors declare no conflicts of interest.
